# Numerical Simulation and Experimental Investigation of Cold-Rolled Steel Cutting

**DOI:** 10.3390/ma11071263

**Published:** 2018-07-23

**Authors:** Jarosław Kaczmarczyk, Adam Grajcar

**Affiliations:** 1 Institute of Theoretical and Applied Mechanics, Silesian University of Technology, 18A Konarskiego Street, 44-100 Gliwice, Poland; jaroslaw.kaczmarczyk@polsl.pl; 2 Institute of Engineering Materials and Biomaterials, Silesian University of Technology, 18A Konarskiego Street, 44-100 Gliwice, Poland

**Keywords:** plastic zone, fracture mechanism, steel sheet, cutting process, Huber–Mises stress, finite element method, microscopic analysis

## Abstract

The paper presents results of the investigations on numerical computations and experimental verification concerning the influence of selected parameters of the cutting process on the stress state in bundles of cold-rolled steel sheets being cut using a guillotine. The physical model and, corresponding to it, the mathematical model of the analysed steel sheet being cut were elaborated. In this work, the relationship between the cutting depth and the values of reduced Huber–Mises stresses as well as the mechanism of sheet separation were presented. The numerical simulations were conducted by means of the finite element method and the computer system LS-DYNA. The results of numerical computations are juxtaposed as graphs, tables, and contour maps of sheet deformation as well as reduced Huber–Mises strains and stresses for selected time instants. The microscopic tests revealed two distinct zones in the fracture areas. The ductile and brittle zones are separated at the depth of ca. 1/3 thickness of the cut steel sheet.

## 1. Introduction

Guillotines are conveniently used for cutting of metal sheets because one can cut not only single sheets, but also bundles. Cutting of bundles is more efficient compared to cutting individual sheets, because it allows many sheets to be cut at a single cutting tool passage. The bundle cutting is used to separate many sheets in order to reach high-quality cut surfaces. The most frequently occurring defects are bends of cut sheet edges, burrs, and vertical scratches. To improve the cutting process, the numerical investigations have been carried out concerning mainly the application of finite element method for numerical simulation of cut sheet separation and experimental research aimed at verifying the obtained results. There are many interesting items in the literature regarding mainly machining [1,2,3,4,5,6,7], but much less papers cover the mechanical cutting on guillotines [8]. Different approaches are used for simulating failure, metal separation, etc. The most important is to select an appropriate FEM model [9,10,11]. Machine deformation can also affect significantly the cutting process [12,13].

It can be concluded that cutting on guillotines is a niche subject appearing in the literature rather sporadically when the problems related to machining processes are discussed. Research concerning cutting of single sheets using a guillotine shear can be found in work [14]. The authors formulated physical models and, corresponding to them, mathematical ones using the computer program LS-DYNA [15]. The obtained results indicate that it is possible to model the process of cutting a single sheet on a guillotine shear.

This paper presents physical models and, corresponding to them, mathematical models in the case of cutting a single sheet on a guillotine intended for bundle cutting, the mechanism of which is different from the guillotine shear cutting. The important difference means that during cutting on a guillotine shear, the blade of a cutting tool simultaneously performs a progressive and angular movement similar to one which is observed during cutting a sheet of paper with scissors. Additionally, the blade of a cutting tool passes through the working surface of the table in the cutting process. However, during cutting of sheet bundles on a guillotine, the cutting tool blade performs only vertical movement and the cutting edge of the cutting tool is parallel to the working table surface [8,16]. It should be mentioned that the surface of the table is horizontal and the blade of the cutting tool, after cutting the last sheet in the bundle, stops on the working surface of the table and then returns to its original position.

For experimental investigations, C75S cold-rolled steel was chosen because it is broadly used in the industry for thin sheets. Cold-rolled strips are gaining popularity in various industries. They are increasingly being implemented through a fairly large number of small-component manufacturers as parts and machine components. In many cases, C75S cold-rolled steel requires mechanical separation, which can be achieved by means of guillotines. The cutting on guillotines has great advantages, therefore it is possible to make the separation without a considerable increase of temperature, which ensures saving of unchanged microstructure unlike in, for example, laser cutting.

The objective of the current paper was to improve the understanding of the mechanism of the cutting process and the influence of selected parameters on the state of deformation as well as on the state of strain and, corresponding to it, stress in bundles of cold-rolled steel sheets being cut. The numerical results were obtained using the finite element method and compared with experimental investigations by means of scanning electron microscopy (SEM).

## 2. Physical Model of the Cutting Process

**Figure 1:**
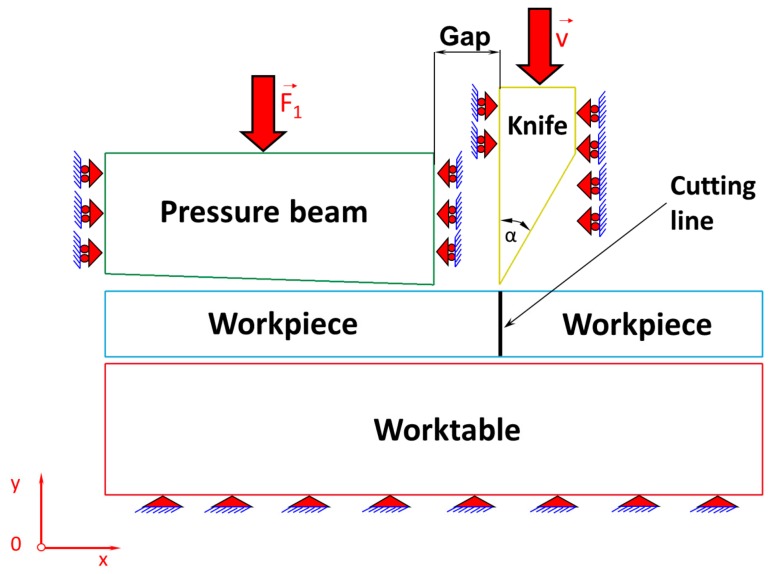
Physical model of a cutting process.

Industrial practice shows that the most common problems of the cutting pieces are: quality of cut surfaces of sheets, arising burrs, and occurrence of numerous defects in the form of vertical scratches on cut sheet metal surfaces and therefore it was decided to develop a model consisting of a deformable sheet and ideally rigid cutting tool, as well as pressure beam and working surface of the table. The physical model of the sheet metal cutting process is shown in Figure 1. On a motionless, perfectly rigid table, a sheet being cut is placed and then it is pressed from the top using a perfectly rigid pressure beam made in the form of a wedge with convergence 1:30 to produce a specific stress concentration on the cutting line. The influence of this concentration of stress in the sheets on the cutting line is extremely important because after exceeding the value of permissible stress, the process of separation of cutting pieces begins. Extensive research on this phenomenon is discussed in detail in [16]. From these scientific investigations, one can conclude that it is easier to cut a single sheet with prestress compared to cutting the same sheet without prestress. It is caused by the fact that on the blade of the cutting tool in the first case, the force acts with a lower value than in the second case. Accordingly, the state of stress in the first case (with prestress) is higher than in the second case (without prestress). The gradual increase in the value of the force on the blade causes an increase of the stress state in the sheet metal, until it reaches such a state which enables the separation of a sheet being cut. There is a small gap between the blade of a cutting tool and the pressure beam. It turns out that the size of this gap has a large impact on the value of the prestress on the cutting line. The numerical simulations indicate that the smaller the gap is, the higher the stresses produced are [16].

Summarising, both the loading force of the pressure beam and the size of the gap affect the value of the prestress state and, consequently, the cutting process. Increasing the load of the pressure beam and reducing the size of the gap increase the prestress state in the sheets, simultaneously decreasing the value of the force acting at the cutting tool blade and required to separate the sheets, which is extremely important on account of extending the life of a cutting tool blade [8].

**Figure 2:**
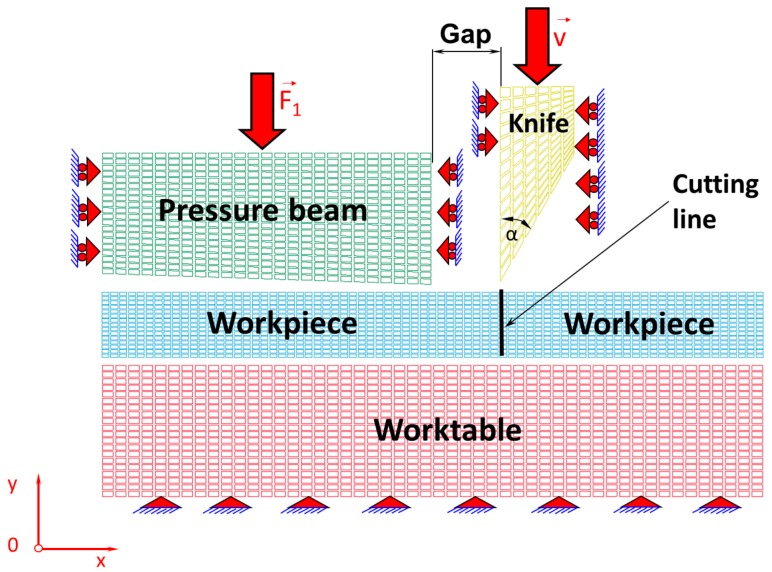
Discretisation into finite elements.

In this work, it is assumed that the cutting tool blade has an apex angle α = 30° and the gap between the cutting tool and the pressure beam is 0.1 mm. The elaborated physical model presented in Figure 1 is divided into finite elements corresponding to a plane state of strain, which is illustrated in Figure 2. The Coulomb’s friction law was used to describe tool–workpiece, pressure beam–workpiece, and workpiece–worktable interfaces friction with the assumed coefficient of friction as for steel. The unilateral constraints in the form of the so-called contact have been imposed on the surfaces of the sheet being cut, worktable, pressure beam, and cutting tool. The contact is based on the condition of impenetrability, namely the condition that two bodies cannot interpenetrate. Impenetrability cannot be expressed as a single equation, so several simplified approaches have been developed by means of the finite element method. In order to enforce a constrained condition on the governing equations, many well-known algorithms have been applied such as: the Lagrange multiplier method, the penalty method, the augmented Lagrangian method, and the perturbed Lagrangian method [17,18,19,20,21,22]. In this paper, the contact penalty method for modelling of the contact between early mentioned surfaces in the cutting process has been exploited.

The cutting process is accompanied by separation of material along a cutting line (Figure 1 and Figure 2). In literature, there are many well-known methods concerning fracture mechanics, which is the field of mechanics that studies the propagation of cracks in materials [6]. It uses approaches of analytical solid mechanics to calculate the driving force on a crack and those of experimental solid mechanics to characterize the material’s resistance to fracture [23,24,25]. It should be mentioned that in modern materials science, fracture mechanics is an important tool applied to improve the performance of mechanical components. It uses the physics of stress and strain behaviour of materials, in particular, the theories of elasticity and plasticity [26] to the microscopic defects found in real materials, in order to predict the macroscopic mechanical behaviour of those bodies [27,28].

In this work, for the criterion of material separation, the extreme strain value at which the sample breaks during its uniaxial stretching was found experimentally. In the model on both sides of the cutting line, corresponding mutual pairs of nodes are created, in which the Huber–Mises reduced strain values are checked for each iteration in the numerical simulation of the cutting process. If the Huber–Mises reduced strain is greater than the limit value strain determined from experimental investigation by the uniaxial tensile test (ɛ_f_ = 0.15), then the corresponding pairs of nodes will be separated on the cutting line. In the opposite case, when Huber’s reduced strains are smaller than ɛ_f_ = 0.15, the nodes will not be separated.

Such elaborated two-dimensional model of the cutting process is correct because it is loaded by forces applied parallel to the plane of the sheet and distributed uniformly over the thickness of the sheet and therefore satisfies the conditions of the plane state of strain [26]. The component of the strain along the normal to the plane strain equals zero. There are infinitely many such planes, and the results of numerical calculations in these planes are repetitive and, as a consequence, comparable with those presented in this work regarding the plane state of strain. Furthermore, it should be noticed that the plane state of strain is simpler in the physical interpretation of the obtained numerical results concerning the modelling of issues related to the mechanical separation of cold-rolled steel sheets and then subsequently cut on the guillotines.

Table 1 presents the data concerning the properties of the components of a physical model, such as the numbers of nodes and finite elements, into which the individual components of the physical model are divided (Figure 2). The numerical calculations were carried out using the computer cluster Ziemowit (http://www.ziemowit.hpc.polsl.pl).

## 3. Material Properties

**Figure 3:**
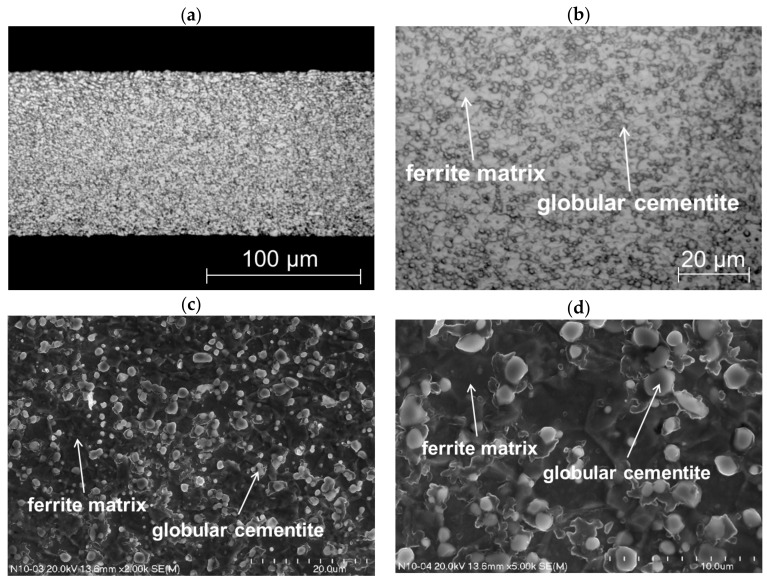
Microstructure of the cut steel shown with the use of light microscope (**a**,**b**) and scanning electron microscope (**c**,**d**).

The material being cut is a C75S cold-rolled sheet steel containing a eutectoid carbon content. Following cold rolling, the steel sheets are submitted to spheroidizing annealing to reduce the hardness to ca. 200 HV. The sheets are homogeneous across their cross sections (Figure 3a). The microstructure is very fine-grained. It consists of ferrite matrix and globular cementite precipitates (Figure 3b). The carbide particles are uniformly arranged within the microstructure. They are separated from each other (Figure 3c). The ferrite grain size is from 3 to 5 μm whereas particle diameters are within a range from 1 to 2 μm (Figure 3d).

**Figure 4:**
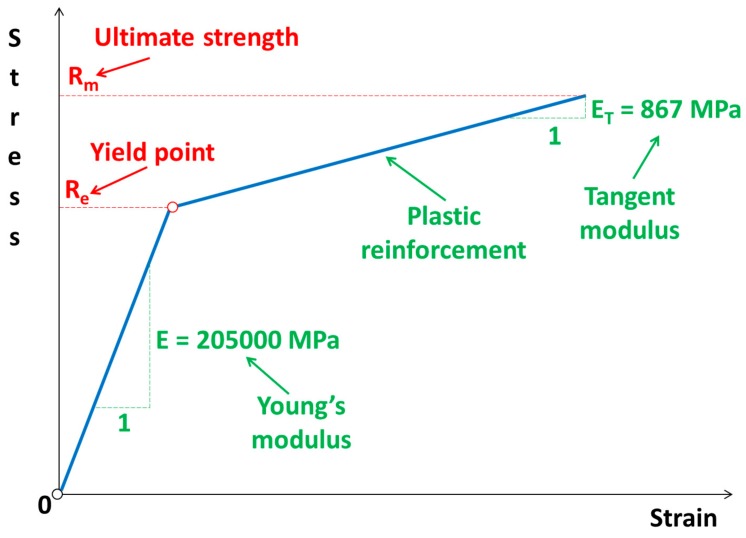
Bilinear elastic–plastic physical material model.

In order to model the cutting process, it is necessary to adopt the appropriate physical model and, corresponding to it, the mathematical one, which will allow the obtaining of satisfactory results. In practice, some simplified material models that give good results are often used. In this work, a bilinear elastic–plastic material model with plastic strengthening is adopted. Detailed material properties are juxtaposed in Table 2 and graphically presented in Figure 4.

## 4. Results and Discussion

**Figure 5:**
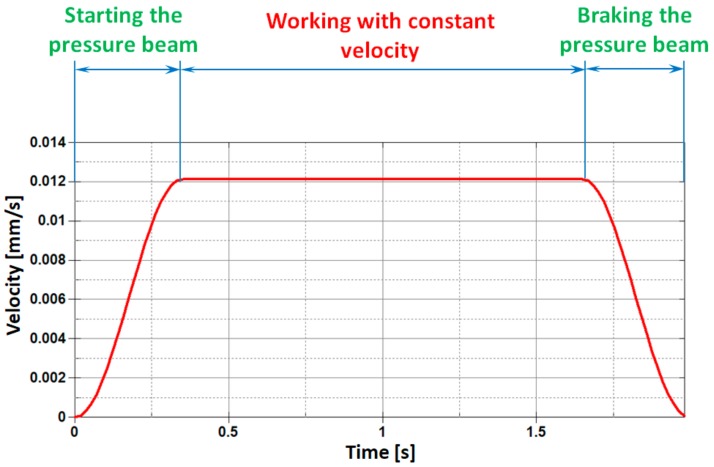
Individual stages of pressure beam motion.

The cutting process has been designed in such a way that it reflects the real conditions of thin steel sheet cutting on the production line in the best possible manner. In the first stage of the process, the sheets are placed on the working surface of the table and then the preloading of the sheet being cut is carried out using a pressure beam. At the initial moment of the cutting process, the pressure beam is released and the movement in vertical direction is realized in order to press the sheet from the top. This movement takes place in three consecutive stages. In the first stage, the pressure beam is released and gently begins to move at a velocity increasing from zero until reaching the maximum constant value of v = 0.012 mm/s, as shown in Figure 5. In the next stage, the pressure beam moves with a constant velocity, and in the final third stage, the pressure beam gently slows down to a halt, simultaneously pressing the sheets with the desired force. For the purposes of this paper, a cutting process with zero pressing force is considered. This does not mean, however, that the pressure beam is unnecessary, but only that it is loaded with a small force, which can be treated as negligible. During the cutting process, it prevents the sheet from sliding or buckling or being put into any possible self-excited vibrations.

In the next stage, the cutting tool is released and starts moving in the vertical direction from zero to a certain limit with constant velocity (v = 0.01 mm/s). After the sheet metal has been cut, the blade of the cutting tool stops at the horizontal working surface of the table and returns to its original position. During the cutting process, the generator of the tip blade is parallel to the horizontal working surface of the table.

**Figure 6:**
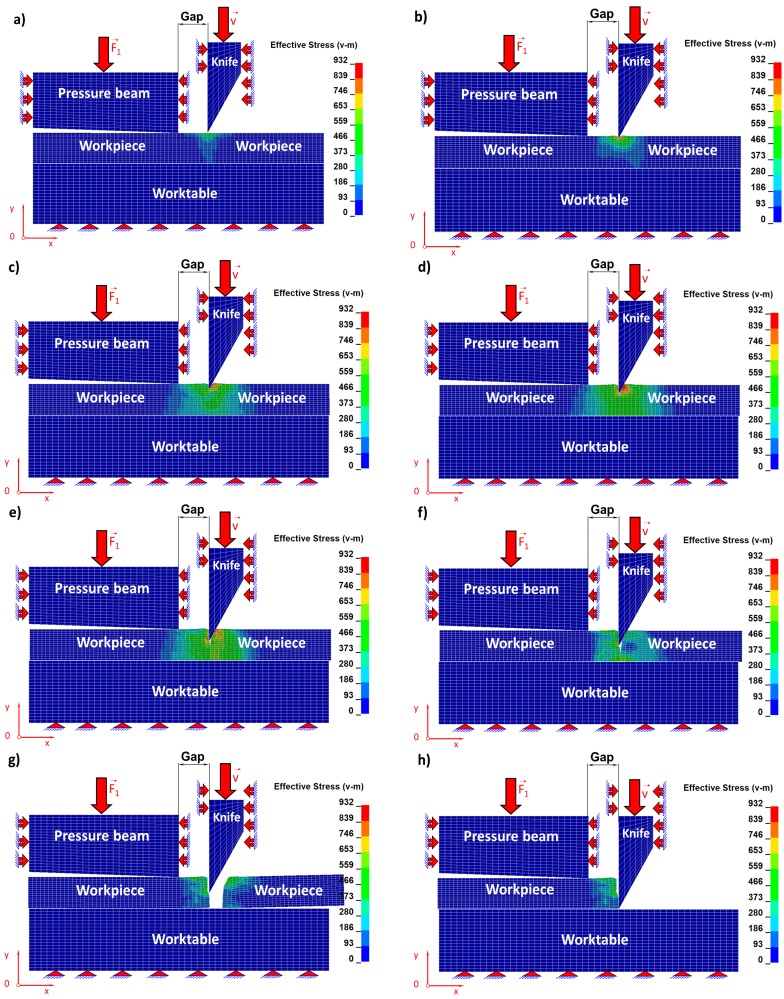
Individual stages of reduced Huber–Mises stresses during numerical simulation of a cutting process for selected time instants: (**a**) t_a_ = 2.07 s; (**b**) t_b_ = 3.0 s; (**c**) t_c_ = 4.0 s; (**d**) t_d_ = 5.0 s; (**e**) t_e_ = 6.0 s; (**f**) t_f_ = 7.1 s; (**g**) t_g_ = 7.2 s; (**h**) t_h_ = 12.0 s.

**Figure 7:**
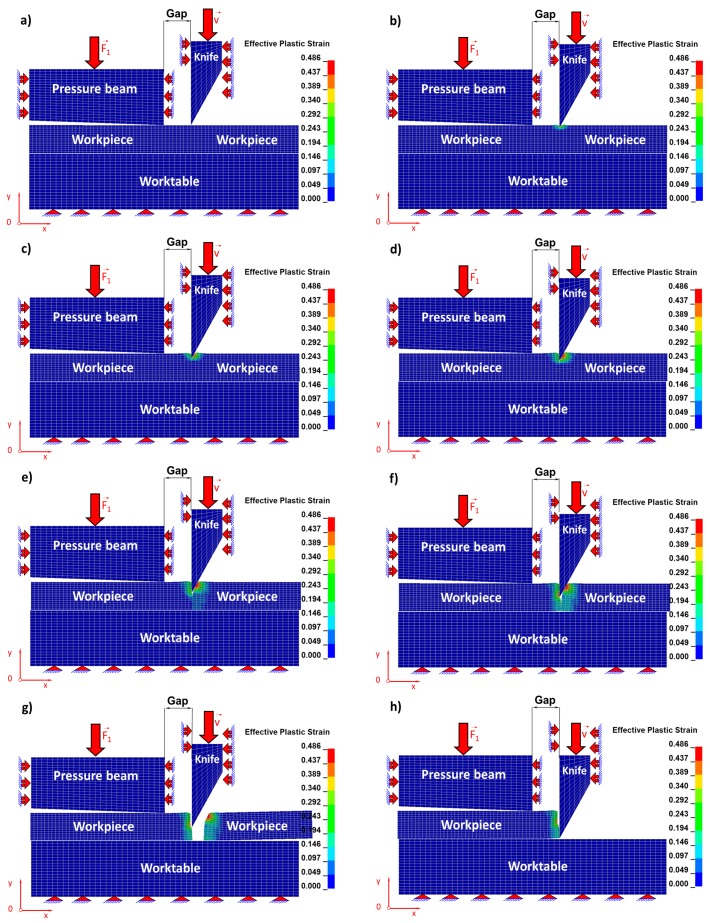
Individual stages of reduced Huber–Mises strains during numerical simulation of a cutting process for selected time instants: (**a**) t_a_ = 2.07 s; (**b**) t_b_ = 3.0 s; (**c**) t_c_ = 4.0 s; (**d**) t_d_ = 5.0 s; (**e**) t_e_ = 6.0 s; (**f**) t_f_ = 7.1 s; (**g**) t_g_ = 7.2 s; (**h**) t_h_ = 12.0 s.

Individual stages concerning the cutting process related respectively to the Huber–Mises reduced stresses of the sheet being cut (Figure 6) and, corresponding to them, states of strains (Figure 7) for selected time instants of the cutting process are juxtaposed and then discussed in detail.

The blade of the cutting tool exerts pressure on the upper surface of a steel sheet and three characteristic zones of the cutting process are formed (Figure 6 and Figure 7). The first is associated with accumulating of stresses and the formation of the elastic zone until the elastic limit is reached (Figure 6a and Figure 7a). The second zone begins when the elastic limit is exceeded and is associated with the formation of a plastic zone. In the second stage, the material from which the sheet is made (Figure 6b,c and Figure 7b,c) is submitted to shearing. In the third zone, the material being cut is separated. This is the most characteristic stage, which can be divided into two consecutive phases. The first initial phase consists in separating of the material being cut as a result of the dominant shearing process with a small participation of stretching (Figure 6d,e and Figure 7d,e) and the second phase consists in separating the cut material as a result of the dominant stretching process, similar to what we observe in the case of uniaxial tensile test but with a vanishing participation of material shearing (Figure 6f,g and Figure 7f,g).

**Figure 8:**
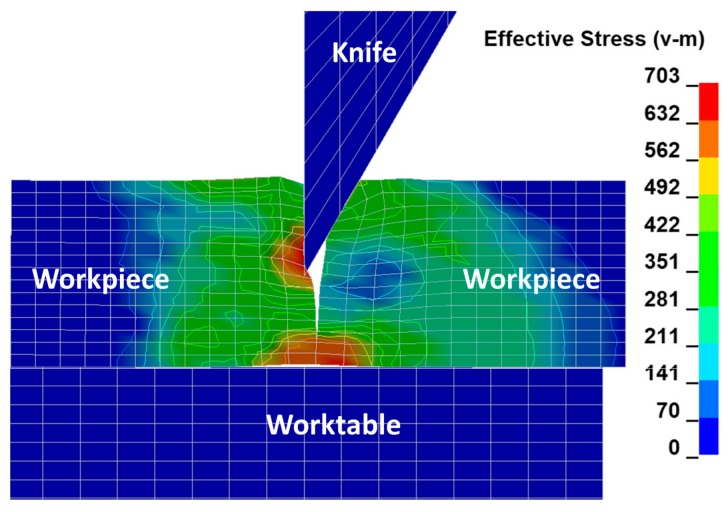
Final phase of a cutting process (cracking of sheet being cut).

**Figure 9:**
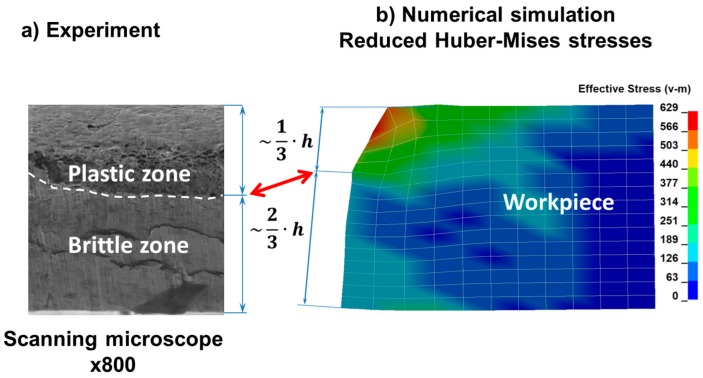
Comparison of the experimental and numerical results: (**a**) view from the forefront of the steel sheet being cut; (**b**) reduced Huber–Mises stresses (the permanent plastic deformation at the left upper edge is visible).

In Figure 8, the final stage of the phase related to the ripping of the sheet being cut consisting in separating the material and creating free surfaces as a result of the progressive cracking process is presented. As a result of observation under the microscope (Figure 9a), it can be seen that the area of the cross section of the steel sheet being cut corresponding to ca. 1/3 of its height measured from the top is subjected to plastic shearing and the remaining cross section area corresponding to ca. 2/3 of the sheet height measured from the bottom is subjected to brittle cracking. The characteristic horizontal crack forming the transition between the plastic and brittle zones is marked with a curved line and shown in Figure 9a. The surfaces of the plastic shearing region are deformed permanently and show large unevenness at the left upper edge (Figure 9b), while those of the brittle fracture region present flat and smooth features without bends or burrs.

**Figure 10:**
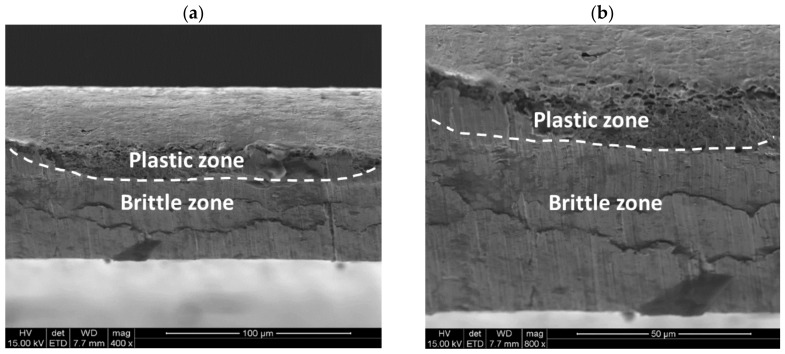
Cross section of the steel sheet being cut showing ductile and brittle zones (**a**) and the magnified part of the cross section showing microscopic details (**b**).

Further microscopic details are visible using the magnifications typical for the scanning electron microscope. Figure 10a indicates that there are two distinct zones (i.e., the upper one has features of plastic deformation and the lower one shows a brittle character). At the higher magnification, it is visible that the upper area shows typical dimples of various size characteristic of ductile fracture (Figure 10b). This part of the cross section is separated from the other one by a curved line. Hence, the separation is not linear. The lower part of the sample shows the typical flat fracture without any dimples. Only brittle walls can be visible. It confirms that the lower part of the steel sheet experiences the brittle cracking.

Summarising the cutting process, one may state that it involves the bending of edges of steel sheets being cut in the plastic shearing zone and the occurrence of burrs and large permanent deformations, while brittle surfaces are characterized by a high degree of smoothness (low roughness), flatness of the cross section, lack of burrs, and thus desirable features of metal sheet surfaces being cut. Then the question arises whether the technological parameters of the cutting process can be selected in the meaning of machine settings to obtain a brittle cross section over the entire height of the sheet instead of 2/3 of the brittle cross-sectional height which is presented in this paper. It seems that the height of the brittle fracture can be increased, while at the same time the height of the plastic zone undergoes reduction. It can be achieved by using classical, evolutionary, or hybrid optimization methods that are a combination of classical optimization methods with genetic or evolutionary ones [29,30,31,32].

In Table 3 and Table 4, the maximum values of component stress and Huber’s reduced stress for selected time instants are juxtaposed. It can be noticed that with the passage of time in the initial stage of the cutting process, these stresses increase, and in the final stage of the process, they decrease, which is the result of the progressive separation of the material being cut.

**Figure 11:**
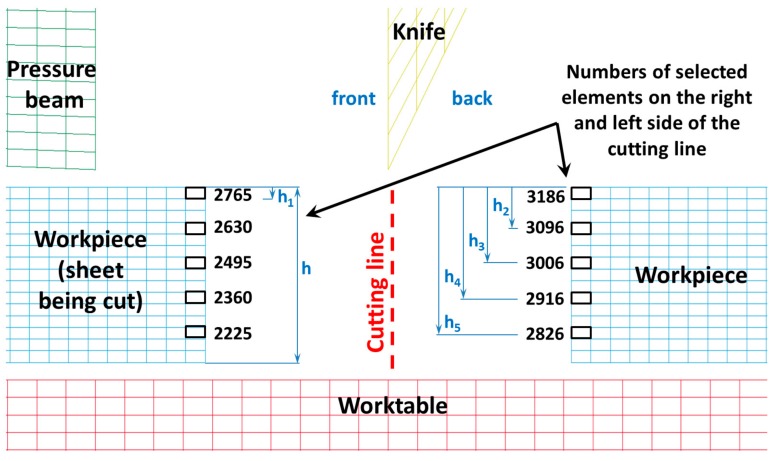
Sheet with selected marked numbers of finite elements accordingly in front of the cutting tool (on the left of the cutting line) and in the back of the cutting tool (on the right of the cutting line).

**Figure 12:**
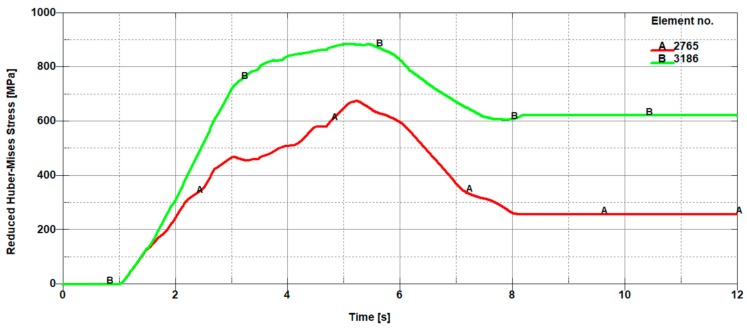
Mean reduced Huber–Mises stresses from the front (A 2765) and from the back (B 3186) of a cutting tool, which correspond to finite element numbers 2765 and 3186, respectively (Figure 11).

**Figure 13:**
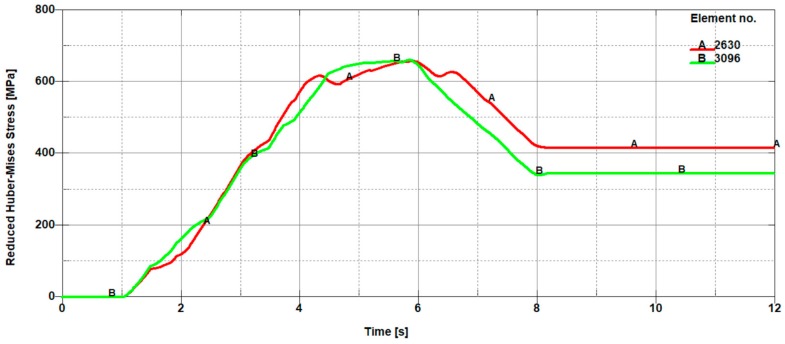
Mean reduced Huber–Mises stresses from the front (A 2630) and from the back (B 3096) of a cutting tool, which correspond to finite element numbers 2630 and 3096, respectively (Figure 11).

**Figure 14:**
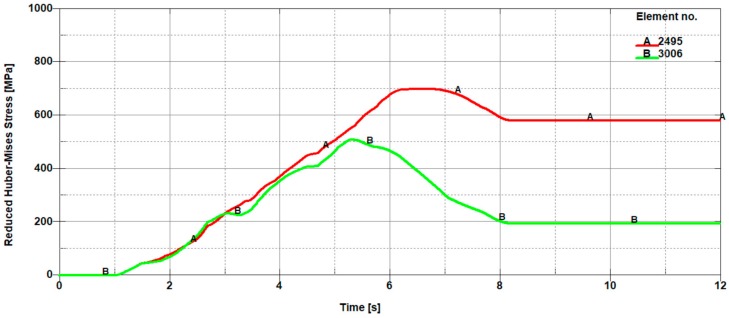
Mean reduced Huber–Mises stresses from the front (A 2495) and from the back (B 3006) of a cutting tool, which correspond to finite element numbers 2495 and 3006, respectively (Figure 11).

In Figure 11, the selected finite elements along with their numbering belonging to the sheet being cut in front of the knife (on the left side of the cutting line) and in the back of the knife (on the right side of the cutting line), depending on the height on which they occur, are presented. In contrast, in Figure 12, Figure 13 and Figure 14, the mean reduced Huber–Mises stresses for several selected heights (h_1_–h_3_) as a function of time for the entire cutting process are juxtaposed. It turns out that at a small height measured from the upper surface of the sheet (h_1_), stresses on the right side of the cutting line are significantly higher compared to the stresses on the left side of the cutting line (Figure 12). The high values of the mean reduced Huber–Mises stress obtained as a result of the numerical simulation exceeding the yield point (R_e_ = 510 MPa) indicate that the sheet edge on the right side of the cutting line is subjected to the large plastic deformations compared to the edge of the sheet on the left side of the cutting line, where the maximum stresses slightly exceed the yield point and therefore the corresponding plastic deformations are insignificant in this case. A similar situation is maintained at a slightly higher depth corresponding to the height h_2_, with the only difference, in comparison to the variant concerning the height h_1_ (Figure 12), is that the difference in stresses on the left and right side of the cutting line is relatively small (Figure 13). The influence of different parameters on the cutting process was the subject of research of Nouari and Makich [2], who presented the analysis of mechanisms involved during the machining process of titanium alloys. They investigated the effect of cutting parameters on the tool wear behaviour and stability of the cutting process. Their results showed that during machining and the action of the cutting tool edge, the workpiece material underwent a strong compression and deformed plastically similarly to the cutting process of cold-rolled steel sheet (Figure 10).

With increasing height of delving of the cutting tool blade from h_2_ to h_3_, there is still a tendency for the occurrence of high stress values in the direct cutting zone exceeding the yield point (R_e_). At the depth corresponding to the height h_3_, there is a characteristic change in the cutting process involving the change of the location of the extreme in the mean reduced Huber–Mises stresses. For the considered height of delving of the cutting tool blade into the material being cut, the mean reduced Huber–Mises stresses change the location of the extreme occurrence. Extreme stress values start to dominate on the left side of the cutting line (in the front of the knife), which was presented in Figure 14. This change can be explained by a variation in the nature of the cutting process.

In the initial phase, the cutting process consisting in the pure shear, which is inevitably accompanied by the occurrence of large plastic deformations, goes into a more complex process involving the simultaneous occurrence of shear with dominant stretching such as can be observed in the uniaxial tensile test. The mechanism responsible for stretching in the cutting process is the blade of the cutting tool in the shape of the wedge with an apex angle of around 30°. The wedge moving with translational motion from the top towards the bottom is the cause of the occurrence of dominant shear and slight stretching in the first phase and of fading shear with the dominant share of tearing in the next phase. The latter state consequently leads to a brittle cracking resulting in the separation of the sheets being cut.

The observed change in the location of extremes first on the left and then on the right side of the cutting line constitutes a certain characteristic state, which is responsible for creating the transition of a so-called bridge between the plastic and brittle state induced by the dominant tearing state, which is illustrated in Figure 8. The change in the location of extremes of the mean reduced Huber–Mises stresses coincides with the curve that forms the boundary between the plastic and brittle zones. At successive depths corresponding to the heights h_4_ and h_5_, one can see that the stresses on the left side of the cutting line are slightly higher than the stresses on the right side of the cutting line. The extreme stress values are only slightly higher than the yield stress, which means that the final phase associated with the separation process of sheets takes place as a result of negligible low shear with a dominant share of stretching accompanied by cracking in the final stage of the cutting process.

## 5. Conclusions

The numerical simulations concerning the mechanism of the cutting process on guillotines by means of the computer program LS-DYNA using an explicit nonlinear dynamic analysis have been conducted. The experimental investigations of C75S cold-rolled steel cutting have been performed on the microstructure and compared with the numerical results obtained by the finite element method. The detailed analysis of the numerical results and metallographic observations allowed the authors to formulate the following conclusions:

- During sheet cutting, the reduced Huber–Mises stresses increase above the limit of steel strength in the direct cutting zone, which causes the material to be destroyed by its separation as a result of the progressive cutting process.
- The stresses in steel sheet grow from zero to the limit of elasticity and then, after exceeding the elastic limit, the plastic zone is formed and the process of material destruction begins along with its separation to the cutting depth of about 1/3 of the sheet height.
- After delving of the cutting tool blade into the metal sheet to a depth greater than 1/3 of its height, the mechanism responsible for separating the material changes. The shear ceases to dominate and stretching domination begins, which causes further progressive destruction of the material by ripping it, resulting in brittle fracture. This stage is advantageous in terms of obtaining sheets with a high smoothness without burrs.
- The stage of plastic shearing causes bending of the edges of the sheets, mainly as a result of large plastic deformations occurring in the first phase of the cutting process. The occurrence of a characteristic line pointing to the border between two areas, plastic and brittle, in microscopic images, as well as the values of the reduced Huber–Mises stresses resulting from numerical calculations, implying the permanent plastic deformation, indicate the correctness of the assumed physical model of the cutting process.

## Figures and Tables

**Table 1:** Juxtaposition of data concerning the components of a physical model.

No	Name of the Part	Kind of Part	Number of Nodes	Number of Elements
1.	Worktable	Rigid	1071	1000
2.	Workpiece	Deformable	1232	1125
3.	Pressure beam	Rigid	546	500
4.	Cutting tool (knife)	Rigid	112	90
**Total Number**			**2961**	**2715**

**Table 2:** Material properties.

No	Name of the Material Properties	Symbol	Value
1.	Young’s modulus	E	2.05 × 10^5^ MPa
2.	Poison’s ratio	ν	0.28
3.	Kirchhoff’s modulus	G	8 × 10^4^ MPa
4.	Tangent modulus	E_T_	867 MPa
5.	Failure strain	ε_f_	0.15
6.	Yield stress	R_e_	510 MPa
7.	Ultimate tensile strength	R_m_	640 MPa

**Table 3:** Juxtaposition of maximum reduced Huber–Mises stress and strain values in a sheet being cut for selected time instants during numerical cutting process.

Figure 6 and Figure 7	Time Instant (s)	Reduced Huber–Mises Stress (MPa)	Reduced Huber–Mises Plastic Strain (-)
(a)	t_a_ = 2.07	512	0.003
(b)	t_b_ = 3.0	717	0.376
(c)	t_c_ = 4.0	817	0.425
(d)	t_d_ = 5.0	913	0.465
(e)	t_e_ = 6.0	874	0.486
(f)	t_f_ = 7.1	720	0.486
(g)	t_g_ = 7.2	622	0.486
(h)	t_h_ = 12.0	622	0.486

**Table 4:** Juxtaposition of maximum constituent stress values in a sheet being cut for selected time instants during numerical cutting process.

Time Instant (s)	Stress along *x* Axis (MPa)	Stress along *y* Axis (MPa)	Shear Stress in *xy* Plane (MPa)
t_a_ = 2.07	60	379	271
t_b_ = 3.0	309	268	358
t_c_ = 4.0	471	404	436
t_d_ = 5.0	625	784	520
t_e_ = 6.0	690	472	497
t_f_ = 7.1	745	644	226
t_g_ = 7.2	472	568	309
t_h_ = 12.0	467	572	251
